# Progressive hemifacial atrophy: a review

**DOI:** 10.1186/s13023-015-0250-9

**Published:** 2015-04-01

**Authors:** Stanislav N Tolkachjov, Nirav G Patel, Megha M Tollefson

**Affiliations:** Mayo Clinic, Department of Dermatology, 200 First Street SW, Rochester, MN 55905 USA; Mayo Clinic, 13400 E. Shea Blvd, Scottsdale, AZ 85259 USA

**Keywords:** Progressive hemifacial atrophy, Facial atrophy, Facial hemiatrophy, Parry-Romberg syndrome, Romberg’s syndrome, Morphea, Scleroderma

## Abstract

**Background:**

Progressive Hemifacial Atrophy (PHA) is an acquired, typically unilateral, facial distortion with unknown etiology. The true incidence of this disorder has not been reported, but it is often regarded as a subtype of localized scleroderma. Historically, a debate existed whether PHA is a form of linear scleroderma, called morphea *en coup de sabre* (ECDS), or whether these conditions are inherently different processes or appear on a spectrum (; Adv Exp Med Biol 455:101–4, 1999; J Eur Acad Dermatol Venereol 19:403–4, 2005). Currently, it is generally accepted that both diseases exist on a spectrum of localized scleroderma and often coexist.

The pathogenesis of PHA has not been delineated, but trauma, autoimmunity, infection, and autonomic dysregulation have all been suggested. The majority of patients have initial manifestations in the first two decades of life; however, late presentations in 6th and 7th decades are also described [J Am Acad Dermatol 56:257–63, 2007; J Postgrad Med 51:135–6, 2005; Neurology 61:674–6, 2003]. The typical course of PHA is slow progression over 2-20 years and eventually reaching quiescence.

Systemic associations of PHA are protean, but neurological manifestations of seizures and headaches are common [J Am Acad Dermatol 56:257–63, 2007; Neurology 48:1013–8, 1997; Semin Arthritis Rheum 43:335–47, 2013]. As in many rare diseases, standard guidelines for imaging, treatment, and follow-up are not defined.

**Methods:**

This review is based on a literature search using PubMed including original articles, reviews, cases and clinical guidelines. The search terms were “idiopathic hemifacial atrophy”, “Parry-Romberg syndrome”, “Romberg’s syndrome”, “progressive hemifacial atrophy”, “progressive facial hemiatrophy”, “juvenile localized scleroderma”, “linear scleroderma”, and “morphea en coup de sabre”. The goal of this review is to summarize clinical findings, theories of pathogenesis, diagnosis, clinical course, and proposed treatments of progressive hemifacial atrophy using a detailed review of literature.

**Inclusion- and exclusion criteria:**

Review articles were used to identify primary papers of interest while retrospective cohort studies, case series, case reports, and treatment analyses in the English language literature or available translations of international literature were included.

## History and nomenclature

First described by Parry in 1825, and Romberg in 1846, this constellation of craniofacial findings was labeled as progressive hemifacial atrophy by Eulenberg in 1871. Other names used to describe this disorder include Parry-Romberg syndrome (PRS), idiopathic hemifacial atrophy, progressive facial hemiatrophy (PFH), and Romberg’s syndrome. Interestingly, evidence of the disease dates back to ancient Egypt with mummies exhibiting craniofacial dysmorphism consistent with PHA [1-11].

## Epidemiology

Studies to determine true incidence and epidemiologic characteristics of PHA have been elusive due to the rarity of the disease, a lack of standardized criteria for diagnosis [12], and the overlapping features of PHA and ECDS [13].

PHA is more often seen in the female sex, and similarly, morphea is typically described as having a female predilection [14]. In one recent series of 32 patients, 66% were female [15]. This is a similar ratio seen in other recent studies, including one with 54 patients, and another with 22 patients [1,13]. Older studies have shown a slightly higher female prevalence. In the aforementioned review of 772 cases, the female to male ratio of 3:1 [16]. This was replicated in a recent global survey of PHA (3:1); however, there may have been some responder bias leading to the higher female prevalence [5].

## Clinical course and associations/complications

PHA usually presents in the first 20 years of life, although, late-onset disease has also been described [4,15,17,18]. A Mayo Clinic study of 54 patients, grouping ECDS and PHA, showed an average age of onset of 13.6 years with a median of 10.5 years and a range of 0.3 to 75 years. These findings have been corroborated by other large reports [1,5,13,17,19].

After initial presentation, the disorder is usually slowly progressive but self-limited [12]. The disease typically “burns out” in 2-10 years before becoming stationary [16,20,21]. While a majority of patients experience halting of the facial atrophy, in the aforementioned global internet survey, 26% of patients reported disease acceleration. 68% of these cases were women and experienced worsening of facial hemiatrophy during pregnancy (9 women) or after childbirth (8 women). Of the progressive disease cases, stress (26%) and surgery (8%) were identified as possible triggers for acceleration [5]. However, the level of disease activity of each responder could not be assessed by the author. The slowly progressive nature of the disease, and at which point patients are on the spectrum of disease activity, must be taken into account to distinguish disease acceleration versus normal evolution.

## Clinical description

**Figure 1:**
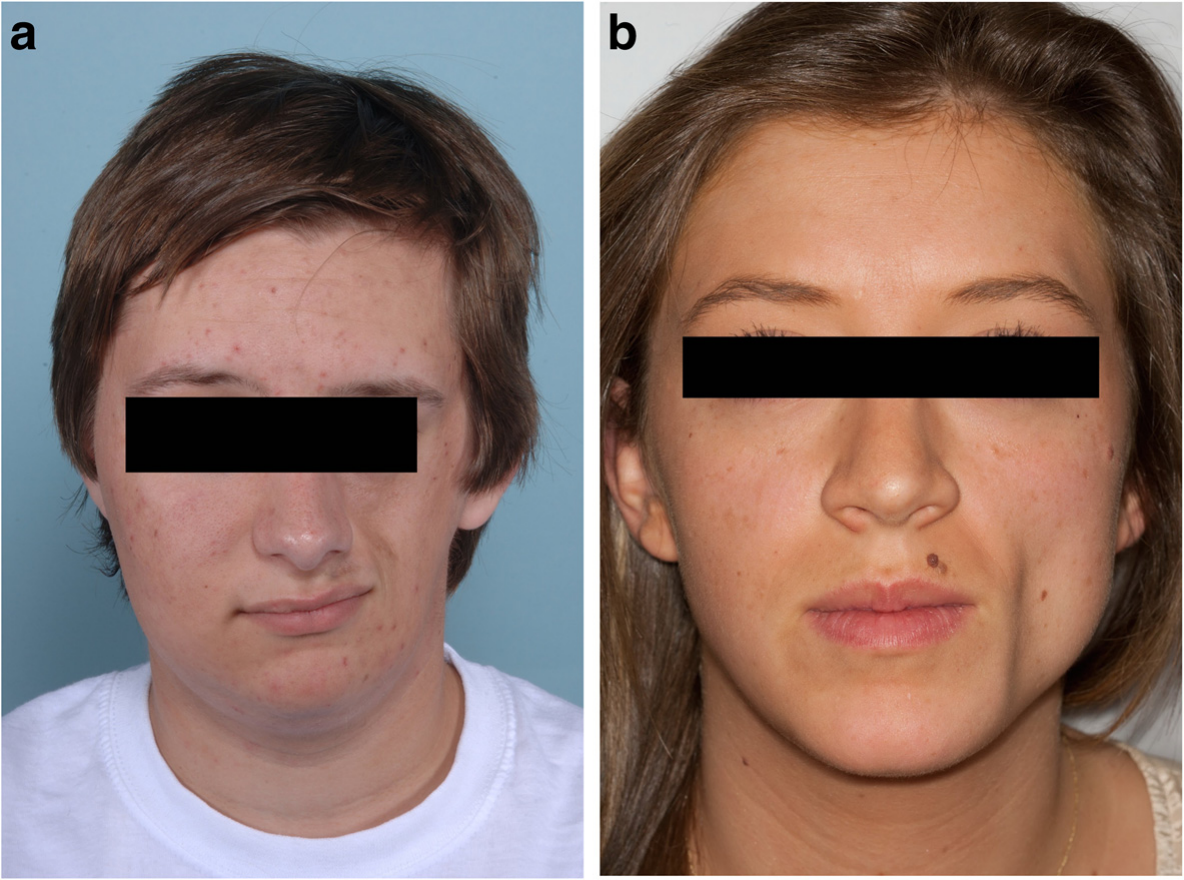
**Progressive Hemifacial Atrophy. a**: Hemifacial atrophy affecting subcutaneous tissue, fat, muscle, and osteocartilaginous structures. **b**: Left-sided sunken appearance secondary to subcutaneous and osseous atrophy.

PHA refers to hemifacial atrophy of the skin and craniofacial tissue inferior to the forehead, typically involving dermatomes of one or more branches of the fifth cranial nerve [12,22]. The atrophy affects subcutaneous tissue, fat, muscle, and osteocartilaginous structures creating a sunken hemiface appearance [12]. (Figure 1a and b) Epidermal cutaneous involvement is minimal, but the tongue, gingiva, teeth, and palate may also be involved [13,16,18,23].

**Figure 2:**
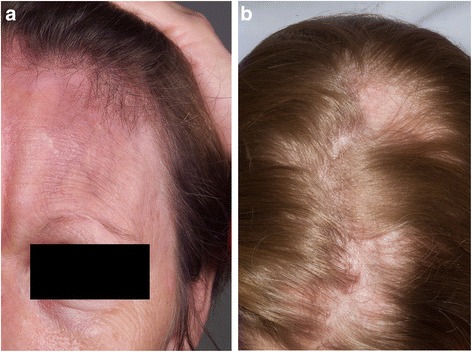
**Morphea En Coup de Sabre. a**: Atrophic shiny plaque with scalp and eyebrow alopecia. **b**: Scalp linear alopecia in a patient with ECDS.

**Figure 3:**
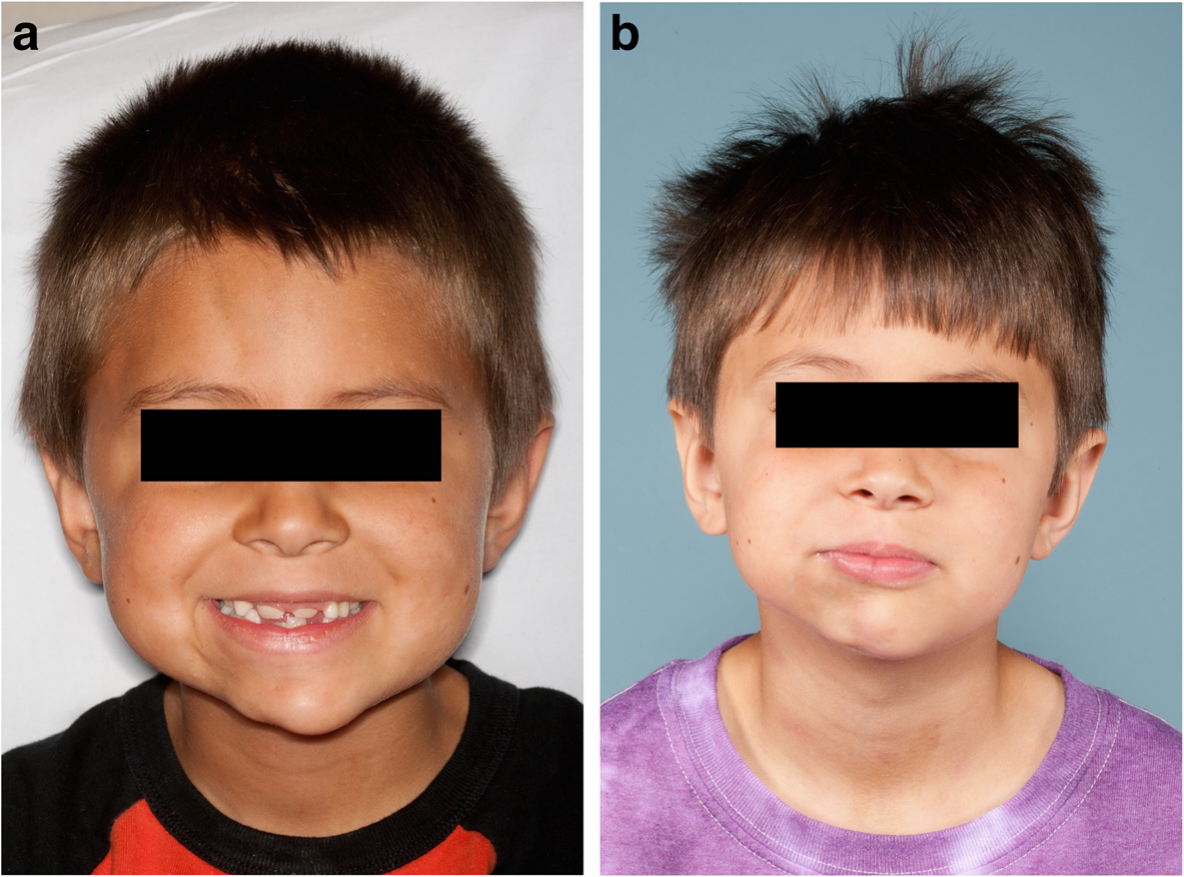
**Morphea ECDS and PHA. a**: Patient demonstrating concurrent right ipsilateral morphea ECDS and PHA. **b**: Same patient with concurrent morphea ECDS and PHA 2 years later.

ECDS manifests as a lesion of linear depression generally located on the frontoparietal scalp or paramedian forehead [24]. It is usually a linear unilateral depression, which may extend below the forehead, involving the nose, medial cheek, and sometimes the upper lip, although rare bilateral cases have been described. Involved skin is hyperpigmented, shiny, firm, and displays alopecia [13,25]. The atrophic shiny plaque may be associated with the scalp or madarosis. Hence, a resemblance of this furrow to a “stroke from a sword”. (Figure 2a and b) While the two may coexist in the same patient, (Figure 3a and b) clinical features are typically used to distinguish PHA from ECDS [12,21,26].

PHA is associated with multiple extracutaneous findings, of which neurologic complications are the most common. Seizures and headaches are the most common neurologic symptoms in patients with PHA. In patients with epileptic activity, simple or complex partial seizures originating in the ipsilateral cerebral cortex are most often seen, and can be refractory to treatment. In patients with other forms of localized and systemic scleroderma, seizures, headaches, and other central nervous system findings may sometimes be seen, but peripheral and autonomic neuropathies tend to predominate [1,7,15,17,27-30].

Cranial neuropathies involving cranial nerves III, V, VI, and VII, have also been described in patients with PHA [19,31-34]. Secondary trigeminal neuralgia has been reported due to impingement of the nerve by destruction of bony structures, as well as vascular inflammation and damage resulting in facial pain that can be chronic and poorly responsive to treatment [35,36]. Additionally, speech may be affected in PHA patients resulting in dysarthria or aphasia [37,38]. Cognitive impairment and an increase in behavioral disorders have also been noted [15,39]. Depending on the degree of atrophy, changes to intracranial tissue and vessels may also result in hemiparesis, dysesthesias, and paresthesias [34,37,40-42].

Vascular damage in the form of infarction, hemorrhage, and white matter hyper-density can be seen in patients with PHA and is most likely due to vascular inflammation [1,41,43]. Dysplastic vessel formation has also been demonstrated in damaged areas of the cerebellum in PHA patients and may also contribute to intracranial findings [44,45]. Vascular damage, dysplastic vessels, and changes in vessel diameter may lead to cerebellar atrophy and production of neurologic symptoms. Some of this may be reversible with treatment [39,46]. Rare intracranial vascular malformations may be seen in patients with PHA and usually consist of dilation of intracranial blood vessels [40,47-49].

Ocular problems may be seen in patients with PHA; these can result in mild visual impairment to blindness [50]. Because of atrophy involving deeper tissue, enophthalmos has been frequently described and often results in loss of function of the orbitalis muscle. Other tissues altered by the atrophic process include the eye itself, the eyelid, and other extraocular muscles. The atrophy may be so severe that restrictive strabismus may result [51-54]. Additionally, patients with PHA have sometimes been noted to have retinal vasculitis, thus further supporting a possible vascular pathogenesis. Less common findings of neuroretinitis, uveitis, papillitis, glaucoma, cataracts, pigmentary changes of the retina and optical fundus, and iris heterochromia have also been demonstrated in some patients with PHA [51,55-61].

Teeth abnormalities and involvement of the mandible and masticatory muscles is frequently seen in patients with PHA. Shorter crowns and roots and crowding of teeth have been seen, as well. Secondary to maxillary and mandibular hypoplasia, chewing, smiling, and speech may be affected, although hemiatrophy and disturbances of other oral structures such as the tongue, lips, salivary glands, and gingiva can also contribute to the development of these symptoms. In those with dental involvement, infection is frequent. Pain secondary to masticatory muscle spasm, temporomandibular joint pain, and locking of the jaw has also been reported [52,62-69].

Skeletal hypoplasia of involved areas of the skull is common, although the clinical significance of this is unknown [52,70]. Atrophy of the extremities, both ipsilaterally and contralaterally, as well as the trunk, has been described in rare cases [12,18,71-73]. Although the reason for this peripheral atrophy is unknown, one such affected patient had normal magnetic resonance angiography in the atrophic limb with only reduction in volume of muscle and fat, making an ischemic or vascular process unlikely in that case [12].

### Relationship of morphea ECDS and PHA

Multiple studies have tried distinguishing PHA and ECDS based on clinical and histopathologic criteria [2,13,19]. Thomas Lehman mentioned two key clinically differentiating factors: paramedian atrophy of PHA without significant induration of the overlying skin and associated atrophy extending down the side of the face with tongue and mandibular involvement. ECDS, in his opinion, is associated with cutaneous induration in the area of the scalp and does not extend below the forehead [74,75]. His disclaimer, that most cases lack clear distinction, is echoed by multiple authors and is consistent with the thought that these entities lie on the same spectrum of disease [2,3].

Some authors have proposed that these two diseases may be differentiated clinically and histologically. In one series of 13 cases of ECDS and 9 cases of PHA, cases were retrospectively evaluated using photos and histopathologic slides [13]. The authors found that the main clinical difference between the two entities was cutaneous sclerosis (present in 8/13 of ECDS and 0 PHA), hyperpigmentation, and alopecia in ECDS, versus total hemifacial involvement and ocular changes in PHA. Histopathologically, however, there was significant overlap as connective tissue fibrosis was seen in all cases with ECDS and in 2/9 cases of PHA. Adnexal atrophy was seen in 11/13 of ECDS and 3/9 of PHA while mononuclear cell infiltrates was present in all ECDS biopsies and 6/9 PHA. Another case series that followed 71 ECDS and PHA patients over a 20 year timespan, found that the clinical morphological pattern of the disease presentation changed progressively such that clear distinctions between the two entities often disappeared over time [3] (Table 1).

**Table 1:** **Comparison of morphea ECDS and PHA**

	**Morphea** ***en Coup de Sabre***	**Progressive Hemifacial Atrophy**
Average Age (years)	10	13.6
Gender (F:M)	2:1 – 3:1	3:1
Clinical Features	-Cutaneous induration/sclerosis	-Paramedian atrophy
	-Scalp to forehead^^^	-No overlying skin induration
	-Hyperpigmentation	-Atrophy may extend down entire face
	-Alopecia (scalp/eyebrow)	
Histopathologic Features~	-Dermal Sclerosis	-Dermal sclerosis
	-Adnexal atrophy	-Fat atrophy
	-Mononuclear cell infiltrates*	-Decrease in adnexal structures
		-Mononuclear cell infiltrates*
Extracutaneous Associations		-Atrophy subcutaneous tissue, fat, muscle, and osteocartilaginous structures
		-Atrophy and deformity of the tongue, teeth, and gingiva
		-Cranial neuropathies
		-Vision loss
		-Seizure disorder

Female (F).

Male (M).

*Histopathologic features depend on disease activity.

^^^May extend below the forehead and involve the nose, medial cheek, and upper lip.

^~^Connective tissue fibrosis, adnexal/fat atrophy, and mononuclear cell infiltrates all seen ECDS > PHA although considerable overlap may be seen.

Duymaz et al. proposed clinical criteria when evaluating a patient with facial atrophy. PHA was suggested when the patient presented with unilateral facial atrophy and lacked previous induration, inflammation, or cutaneous atrophy and sclerosis. Scleroderma ECDS was favored if unilateral band-like changes of sclerosis with hyperpigmentation in the frontoparietal area with associated induration was noted. Involvement of the area above the eyebrow without extension inferiorly, with cutaneous sclerosis and marked deformity, was noted to better describe ECDS [12]. However, histopathologic findings were not examined.

Often, clinical findings of both are present in the same individual [1,17,43,74,76]. In fact, 28 to 42% of patients have been reported to have coexisting ECDS and PHA [1,17,76]. In one review of 235 patients with localized scleroderma at Mayo Clinic from 1923 to 1954, facial hemiatrophy was associated with 41.3% of the 29 localized scleroderma cases involving the frontal, frontoparietal, or facial regions [77]. A large literature review of 772 cases mentioned ECDS as a potential initial presentation in some cases. It is possible that some of these patients may have had coexisting disease or only ECDS [16]. In another evaluation of 58 cases of ECDS, 20 developed signs of PHA [2]. Additionally, progression or transition of ECDS into PHA in the same physical location has also been illustrated in a handful of cases [2,3].

In another large series of ECDS and PHA patients, the authors concluded that their findings support these two entities as being on a spectrum of disease. 71.4% of the 28 patients with PHA in their study had facial sclerosis, with 53.6% having en coup de sabre lesions. Additionally, they found that of the 41 patients with ECDS, 36.6% also had PHA. Histologically, the biopsy specimens of PHA patients who did not have cutaneous sclerosis showed findings consistent with morphea [1]. Several prior studies had similar results [17,78].

In summary, while there may be some features that clinically distinguish ECDS from PHA, clinical and histopathologic findings may often overlap in the same patient, thus supporting the belief that ECDS and PHA fall on the same spectrum of disease.

## Diagnosis

The diagnosis of PHA is a largely clinical one, supported by other findings such as histopathology and imaging. No universal diagnostic criteria are accepted. As discussed above, the characteristic clinical features of PHA which allow for its diagnosis is the presence of unilateral idiopathic facial atrophy, typically involving the lower face, without significant epidermal change. Deeper involvement of bone, teeth, tongue, and gingiva may also be present (Table 1). Patients are typically classified as having ECDS if they demonstrate linear scleroderma of the frontoparietal scalp with involvement of medial or paramedian forehead with possible extension of the sclerosis onto the scalp, nasal sidewall, and maxilla [1]. The cutaneous sclerosis, with possible hyperpigmentation and alopecia of ECDS, has been suggested as a potential differentiating factor between PHA and ECDS in those who desire to distinguish between them [13]. However, the boundaries between these entities are blurred as seen in patients with cutaneous sclerosis developing more significant atrophy than typically seen in ECDS, and conversely, those with atrophy without significant epidermal change, going on to develop cutaneous sclerosis [3].

Histopathology of affected areas in PHA, with or without clinical findings of a sclerotic process, shows homogenized dermal sclerosis, fat atrophy, decrease in adnexal structures, and perivascular plasma cells and lymphocytes [1,13,79] (Table 1). On the other hand, biopsy specimens in ECDS have been noted to show hyperpigmentation, alopecia, and cutaneous sclerosis. Atrophy of epidermis, dermis, adnexal structures and subcutaneous tissue has been seen in both PHA (with and without dermal sclerosis) and ECDS. More specific changes may be seen in biopsy specimens of patients with PHA including: sparse to exuberant lymphocytic infiltrates in the dermis, and degenerative changes of vascular endothelium have also been noted in some studies [1,2,13,17,78,80,81]. Hence, biopsies often may not be helpful in the diagnosis of these entities. Since skin biopsy is infrequently done in cases of PHA, photographical records should be maintained and followed throughout the duration of follow-up. As PHA slowly progresses until the disease plateaus, photographical evaluation is helpful in following the disease course. Additionally, if rapid changes in skin induration or overall clinical presentation are noted, a biopsy may be considered. Electroencephalogram (EEG) changes may be seen in patients who have a known seizure disorder and also in those who do not. EEG findings may or may not be associated with intracranial structural or imaging changes [7,30].

Laboratory work up of patients with PHA, as well as other types of localized scleroderma, tends to be largely unrevealing. A positive anti-nuclear antibody is the most common laboratory abnormality, with approximately 25-52% of patients having an elevated titer [17,22,23,78]. Serology for rheumatoid factor, anti-Scl-70, C-reactive protein (CRP), anti-dsDNA antibody, extractable nuclear antigen screening, genotyping for HLA-B27, and anti-cardiolipin antibodies are rarely abnormal and of limited value in PHA patients. Rheumatoid factor has been shown to be elevated in localized scleroderma with extracutaneous involvement and those with arthritis. However, it is currently unknown whether any PHA patients regularly demonstrate an elevated rheumatoid factor [22,23]. Peripheral eosinophilia and an elevated erythrocyte sedimentation rate have also been reported in association with localized scleroderma, but not with PHA [22,23].

Ultrasound can be used to detect presence of sclerosis, monitor disease activity, and treatment progress by measuring dermal thickness and echogenicity of the affected areas. Color Doppler ultrasound has the added benefit of measuring dermal blood flow, an increase of which indicates active disease. In patients with PHA, assessment of the ipsilateral parotid gland and salivary glands may also reveal increased blood flow and a hypoechoic appearance, indicating inflammation of the glands and active disease [82-84].

**Figure 4:**
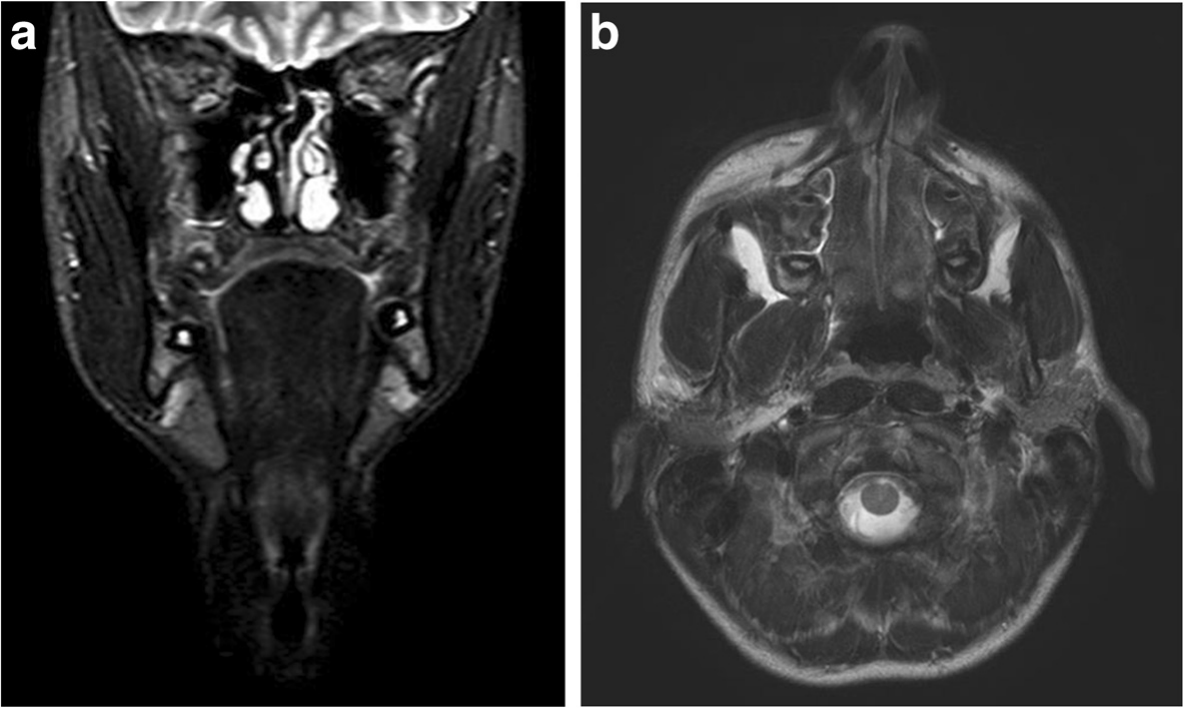
**MRI of Progressive Hemifacial Atrophy. a**: Coronal view from MRI demonstrating left hemifacial atrophy of soft tissue and osseous structures. **b**: Axial view from MRI demonstrating left hemifacial atrophy of soft tissue and osseous structures.

Due to the frequency of neurologic complications in PHA, baseline imaging may be performed, particularly in those patients that have neurologic symptoms. Typical findings on MRI show hyperintense white matter lesions on T_2_-weighted sequences. These are most prominent ipsilaterally but are often seen bilateral despite lack of skin involvement on the contralateral side. (Figure 4a and b) These findings often do not progress despite progressive skin and skeletal involvement. Cerebral atrophy, encephalomalacia, and cystic degeneration have been seen on intracranial imaging of patients with PHA [37]. Computed tomography (CT) scanning may also be used, but is less useful for evaluation of brain parenchyma than MRI. Typically, calcifications and changes in density of white matter can be seen on CT [15,30,85].

Whether or not abnormalities are demonstrated on MRI, not all patients will have corresponding clinical neurologic findings. Additionally, epileptic activity may occur prior or in the absence of any abnormal findings on MRI [30]. Skin findings are also not predictive of CNS involvement [15,19,85]. Because a direct correlation with CNS symptoms, skin findings, and MRI is not always demonstrated, clinical evaluation on an individual case-by-case basis is necessary to determine the need for imaging, whether at baseline, or in follow-up [30,37,85].

Clinically silent white matter findings do occur [86]. Determining the significance of these may be challenging. Single-photo emission CT (SPECT) may be a unique way to test the regional blood flow in the presumingly involved area, as regional blood flow hypoperfusion in the parietooccipital area of the affected hemisphere has been described in PHA patients with temporal lobe epilepsy [87]. Conversely, relative increases in regional cerebral blood flow (CBF) of affected cortical hemisphere on SPECT may be associated with to preserved cortical function and normal psychomotor development [86]. Even if extensive white matter abnormalities are demonstrated on MRI, preserved cortical function may be demonstrated by SPECT, proton MR spectroscopy (^1^H-MR spectroscopy), and diffuse tensor imaging (DTI) [15]. These findings were confirmed in an analysis of 19 PHA and 7 ECDS patients with MRI, angio-MRI, and SPECT for CBF [19]. In this study, the authors found that CNS involvement in patients with PHA may be seen irrespective of when cutaneous indurations were first noted clinically, and a diagnosis of PHA was made. Furthermore, the SPECT may be abnormal in PHA and ECDS whose MRI findings were unrevealing [19]. While observation of CNS symptoms and initial evaluation with MRI when indicated are often favored, the addition of a CBF analysis such as SPECT may give additional detail on the overall CNS involvement and prognosis in this patient population.

If there is a suspicion of a seizure disorder, electroencephalography may be done. In those patients with EEG abnormalities, findings are usually ipsilateral to the clinical involvement and most commonly show localized activity [1,30,88].

As discussed above, ocular manifestations of PHA can be potentially threatening to vision and are common. Ophthalmology should therefore be consulted for evaluation of vision, restrictive strabismus, ocular muscle strength, glaucoma, and inflammatory pathologies associated with the eye and PHA [50,51,61]. Ocular imaging has demonstrated retinal edema and optical disc swelling in some patients, which likely also contributes to visual impairment and loss [89].

Dental involvement in PHA is common, making early evaluation critical in reducing deformity and related complications to the teeth, mandible, maxilla, and other oral structures. Serial panoramic radiographs and photographs can be useful in monitoring disease progress until there is stabilization of disease. This can also assist in planning for surgery and orthodontic appliances [71,90].

## Etiopathogenesis

A leading theory is that PHA is an autoimmune disorder. This is supported by the presence of other autoimmune conditions such as systemic lupus erythematosus (SLE), generalized myopathy, and rheumatoid arthritis (RA) in some patients with PHA [91-97]. Additionally, 17% and 10% of survey respondents with PHA to a survey described a medical history of vitiligo and thyroid dysfunction, respectively [5]. Other autoimmune conditions seen in these responders were inflammatory bowel disease (5%), RA (4%), ankylosing spondylitis (2%), and SLE (2%) [5]. The possible autoimmune etiology is also supported serologically due to the frequent presence of anti-nuclear antibodies, although serologies are of limited use in diagnosis, as discussed above.

Another leading theory in the pathogenesis of morphea form disorders such as PHA is that of vascular dysfunction. Vascular changes may invoke collagen production and extracellular matrix proliferation seen in morphea and systemic sclerosis [91]. It has also been proposed that ECDS and PHA may be related to a neuro-vasculitis [2,46,47,74]. A study of 41 PHA patients with light microscopy performed on 19 tissue specimens and ultrastructural analysis on 6 patients led to the theory that lymphocytic neuro-vasculitis caused by chronic cell-mediated vascular injury and incomplete endothelial regeneration along branches of the trigeminal nerve is central to the pathogenesis of PHA [81]. While not proven, this may contribute to the pathogenesis of this disorder.

There have also been reports of PHA following injury leading to a possible trauma-induced hypothesis [98,99]. In fact, many of the studies focusing on PHA and ECDS have noted trauma in a significant cohort of their study population. The large survey found 27% of responders had a childhood head injury; however, only 12% of responders had injuries that seemed relevant to the authors [5]. Similarly, Sommer et al. found 33% had a preceding injury including sclerotic lesions after a hematoma and secondary to an insect bite [17]. Caution must be used when interpreting this data, however, as these are relatively common occurrences, and there may be a fair amount of responder bias.

Infectious etiologies have also been proposed in the pathogenesis of PHA. It has been suggested that a Bell’s palsy or herpes zoster in the trigeminal distribution may be associated with PHA; however, studies have not been able to corroborate this theory [100]. The coexistence of *Borrelia burgdorferi* antibodies and Lyme infection have been described in European patients with morphea; however, recent studies, in the United Kingdom, United States, and Turkey have not supported this association [101-105]. Of the 12 German cases of PHA reviewed by Sommer et al, none had antibodies to *Borrelia* [17]. A recent study of 21 PHA patients and 6 with ECDS, along with 21 matched controls in Mexico did not show an association between Anti-Borrelia IgG antibodies and PHA, although Lyme disease is uncommon in this geographic location [106]. Other studies from various locations in North America also failed to show a direct association between Borrelia and morphea [107-111].

Lastly, sympathetic hyperactivity and dysregulation has been proposed as a potential pathogenic mechanism in the development of PHA [18,112,113]. A captivating animal experiment by Resende et al. followed cats, dogs, and rabbits for one year after ablation of the superior cervical ganglion at the age of 30 days old. The authors found the altered animals to have evidence of localized alopecia, corneal ulceration, keratitis, strabismus, enophthalmos, ocular atrophy, hemifacial atrophy and slight bone atrophy on the side of the sympathectomy, all potential findings seen in PHA patients [114]. PHA was also induced following a similar experiment in a rat model in 1960 [115]. These associations, however, have not been demonstrated in human patients.

With the current body of evidence, an acquired etiology to PHA is accepted, but familial cases have also rarely been described [113,116,117]. The aforementioned global survey of individuals with PHA found six patients (3%) had family members with facial asymmetry, with only one of these six responders with a first degree relative demonstrating involvement [5]. Autosomal dominant inheritance with incomplete penetrance has been suggested, but no support for this hypothesis has been provided to this point [118,119]. Hence, genetic counseling is recommended in individual cases of PHA. The lack of an etiology consensus and explanation of pathogenesis of PHA also make a defined pattern of inheritance unlikely. In summary, a distinct etiology of PHA is still elusive; however, a combination of autoimmune, vascular, and autonomic dysfunction is likely.

## Differential diagnosis

The differential diagnosis of PHA includes other forms of juvenile localized scleroderma, Rasmussen encephalitis, Barraquer-Simons syndrome, congenital hemiatrophy, and primary hemifacial hypertrophy.

Rasmussen encephalitis (RE) presents with unilateral cerebral hemisphere destruction with intractable focal seizures and progressive neurologic deficits. Pathology from the involved cerebral tissue shows chronic inflammation [120]. The similar ages of onset, unilateral characteristics, and epilepsy occurrence may account for the multiple reports of associations of RE and PHA and the difficulty of differentiating these processes [41,120-124]. In fact, some authors suggest an overlap in the disorders, especially in the cases of PHA that are complicated by seizures [41,120]. Patients with RE alone usually present with hemiparesis and intractable seizures [123]. The clinical hemiatrophy has only been described in PHA patients with coexisting RE or ECDS [41,120,122,123,125]. Additionally, brain biopsies of RE show distinct findings of neuronal loss, reactive blood vessels, and a pale background with neutrophils and chronic inflammatory cells [123]. Brain biopsies are not typically done in PHA. The clinical, and if needed, histopathological differences separate these two syndromes; however, coexistence of RE and PHA is possible.

Baraquer-Simons syndrome is an acquired partial progressive cephalothoracic lipodystrophy that presents with a gradual onset of symmetrical bilateral subcutaneous fat loss from the face, neck, upper extremities, thorax, and abdomen but sparing the lower extremities. Central nervous system findings of deafness, epilepsy, and intellectual disability have also been described [126]. The bilateral nature of this disease and systemic involvement of the kidneys may differentiate these processes. However, associations of Baraquer-Simons lipodystrophy and scleroderma are also described [127].

Hemifacial hypertrophy, formerly termed primary hemifacial hypertrophy, is a rare asymmetric enlargement of half of the head without enlargement of other body parts [128,129]. While there is unilateral face enlargement instead of atrophy as seen in PHA, this disorder may be considered in the clinical differential diagnosis of an asymmetric unilateral facial deformity.

Other clinical mimickers of PHA are fat necrosis, whether from infection such as bulbar poliomyelitis [130], trauma, or connective tissue disease, and congenital deformities such as “wry neck” [131]. Congenital hemiatrophy has been used interchangeably with PHA; however, it is likely a different process [16,132].

## Treatment

Treatment for PHA can be challenging. The primary aim is to stop the active disease process. Methotrexate (MTX) is the standard therapy for active disease. MTX dosing is not standardized and ranges from 0.3-1 milligrams/kilogram/week (mg/kg/wk) with a maximum dose of 25 mg weekly in either an oral or injectable form. The MTX is often combined with oral prednisone over the first three months due to the fact that the methotrexate has a delayed effect on inflammation and fibrosis. Most regimens call for two months of prednisone at 1 mg/kg/day with a taper to be done during the third month. Pulsed high dose IV methylprednisone has also been explored using 1000 mg for three days monthly for 6 months. The goal with this approach is to gain the anti-inflammatory effects of corticosteroids without the large side-effect profile. A long course of therapy is typically required as relapse is frequently seen with shorter courses of therapy. The specific length of therapy required to reduce relapse is unknown, and likely varies from patient to patient. Current evidence supports a 12-24 month course of methotrexate being most effective in inducing prolonged remission [1,23,83,132-136].

Isolated case reports of other immunosuppressive agents such as mycophenolate mofetil, cyclosporine, and cyclophosphamide have shown variable success in patients who have failed treatment with MTX [135,137,138]. The use of antimalarial agents has also been described with some efficacy in a limited number of cases [1]. Ultraviolet-A (UVA) and psoralen combined with ultraviolet A (PUVA) therapy have been shown to be effective in treatment of localized scleroderma [139,140]. PUVA has been reported to arrest disease activity in isolated reports of PHA [103,141].

Surgical treatment for PHA often requires a multi-specialty approach with repeated procedures, depending on degree of involvement. The therapeutic goal of surgery for PHA patients is to minimize psychosocial effects, and to correct the appearance and function of involved facial structures [90,142-145]. Timing of surgical intervention in patients with PHA has been debated. Most experts recommend that procedural therapy be delayed until disease progression has halted or plateaued in order to avoid multiple surgeries as defects progress and because of the need for a stable skeletal foundation [146-152]. However, others argue for earlier intervention despite active disease due to the psychosocial difficulties facing patients with PHA, promotion of normal development of facial structures, and because most patients with PHA require multiple surgeries [144,145]. Ego-dystonic feelings, loss of confidence due to appearance, and bullying are the primary reasons behind pursuing surgical therapy [145]. In one study patients who had earlier surgical intervention had higher satisfaction scores [144], however the utility and timing of surgical procedures must be evaluated on an individual basis.

If surgical therapy is indeed delayed until disease burns out, proper mandibular development and parallelism of facial planes can be guided with orthodontic rehabilitation and orthodontic appliances [142,143]. For mild to moderate atrophy, fat grafting, as well as lipoinjection and other soft tissue fillers, can be employed [142,144,149,153,154]. For more severe atrophy, a combined approach of skeletal and soft tissue augmentation is often recommended. Bone paste cranioplasty, autologous fat infections, dermal fat grafts and adipofascial flaps can also be used to correct large volume atrophy [144,145,155]. Additionally, the use of bone grafts and biocompatible porous polyethylene implants can be used to correct skeletal deformity [145,154,155]. Eyebrow lifting, Z-plasty, lip repair, nasal reconstruction, eyebrow repair, face-lift, lip augmentation, hair transplant, and other adjuvant procedures can also be used to create a better cosmetic outcome [144,145,155].

## Conclusion

Progressive hemifacial atrophy, or Parry-Romberg syndrome, is a slowly progressive and self-limited dysplasia causing unilateral craniofacial atrophy. The close association with morphea en coup de sabre has been investigated, but no consensus on the pathogenesis of PHA is available. We reviewed literature addressing PHA and summarized the relevant findings.

## Limitations

Since the review focuses on English literature, several large studies have been left out including 100 cases from Mussinelli, Magri, and Origlio [156,157], and large German reviews by Möbius in 1895, [158] Marburg [159], and Cassirer in 1912 [160] were excluded. Multiple international case series were reviewed by Roddi et al., in 1994 [21], Archambault and Fromm in 1932, [18] Wartenberg in 1945 [75], and Blair O. Rogers in 1964 [16]. These publications were reviewed in detail.

### Consent

Written informed consent was obtained for the publication of this report and any accompanying images.

## Abbreviations

(PHA): Progressive Hemifacial Atrophy
(ECDS): *morphea en coup de sabre*
(PFH): Progressive facial hemiatrophy
(PRS): Parry-Romberg syndrome
(MRI): Magnetic resonance imaging
(RA): Rheumatoid arthritis
(SLE): Systemic lupus erythematosus
(ANA): Autoantibodies against nuclear
(RF): Rheumatoid factor
(RE): Rasmussen encephalitis
(SPECT): Single-photo emission computed tomography
(^1^H-MR spectroscopy): Proton MR spectroscopy
(DTI): Diffuse tensor imaging
(CBF): Cerebral blood flow
(CNS): Central nervous system
(MTX): Methotrexate
(mg): Milligrams
(kg): Kilogram
(wk): Week
(UVA): Ultraviolet-A
(PUVA): Psoralen combined with ultraviolet A
(MEDPOR) (trade name): Biocompatible, porous polyethylene implants
